# Impact of Celiac Disease on the Quality of Life of Children: Analysis From a Moroccan Cohort

**DOI:** 10.7759/cureus.89539

**Published:** 2025-08-07

**Authors:** Chaimae N'joumi, Amal Hamami, Aziza Elouali, Abdeladim Babakhouya, Maria Rkain

**Affiliations:** 1 Department of Pediatrics, Mohammed VI University Hospital, Faculty of Medicine and Pharmacy, Mohammed I University, Oujda, MAR

**Keywords:** cddux score, celiac disease, chronic disease, gluten-free diet, quality of life

## Abstract

Objective: Celiac disease (CD) requires a lifelong gluten-free diet, which impacts the health-related quality of life (HRQoL) of children. To better understand this impact, our study evaluated the HRQoL of children diagnosed with CD and followed at the University Hospital Center (CHU) of Oujda using the Arabic-validated version of the Coeliac Disease Dutch Questionnaire (CDDUX).

Methods: A cross-sectional study included 49 children with CD, aged 8 to 18 years, and their parents. The CDDUX assessed three subdomains of HRQoL: "gluten-free diet," "communication," and "living with CD." Sociodemographic and medical data were collected and analyzed using Statistical Package for Social Sciences (SPSS) version 25 (IBM Corp., Armonk, NY).

Results: The participants were primarily from urban areas (63%) and mostly female (59%), with an average age of 12.5 years. Most children had been on a gluten-free diet for 1-4 years and were diagnosed at an average age of 7.5 years. Unstable family situations were reported in 23% of children, and 18% of children had associated chronic conditions such as type 1 diabetes or hypothyroidism. The results showed that children and parents had differing perceptions, particularly regarding the social and emotional impact of CD. Parents rated HRQoL lower (39.3±14.9) than the children (42.46±17.2; p=0.01). Children living in rural areas or from unstable family backgrounds reported lower scores.

Conclusion: The HRQoL of children with CD is influenced by sociodemographic and family factors. Social and educational support, particularly for rural and economically disadvantaged families, is essential to improving the perception of HRQoL in children with CD.

## Introduction

Celiac disease (CD) is a chronic autoimmune condition that occurs in genetically predisposed individuals following the ingestion of gluten. It is characterized by villous atrophy in the small intestine [1]. Gluten is a protein complex found in commonly consumed cereals such as wheat, rye, and barley. The clinical presentation of CD is highly variable, ranging from severe symptomatic forms to completely asymptomatic cases. Diagnosis is typically based on serological testing and confirmed by intestinal biopsy [2]. CD may also be associated with other comorbid conditions [3].

To date, the only available treatment is a gluten-free diet (GFD). However, strict adherence to this diet requires significant lifestyle changes, which can impact patients’ quality of life (QoL) [4]. The World Health Organization (WHO) defines QoL as “an individual’s perception of their position in life, in the context of the culture and value systems in which they live, and in relation to their goals, expectations, standards, and concerns” [5].

In children with CD, the social and emotional impact of a GFD can be substantial. Feelings of being different or stigmatized, anxiety about cross-contamination, and dietary restrictions negatively affect their overall well-being [6].

In recent years, the medical community has shown growing interest in how patients perceive the impact of chronic illnesses and their treatments on health. Health-related QoL (HRQoL) is recognized as a multidimensional concept that encompasses physical, emotional, social, and cognitive aspects. It emphasizes patients’ subjective experiences and perceptions of their well-being and daily functioning, rather than relying solely on objective measures [7]. In the context of CD, this subjective dimension is particularly important, given the often subtle nature of symptoms and the profound effects of a strict dietary regimen on daily life and social interactions.

Thus far, only a few studies have explored HRQoL in children with CD, with findings often inconsistent due to the use of varying measurement tools [8-13]. The Coeliac Disease Dutch Questionnaire (CDDUX), developed in the Netherlands in 2008, was the first disease-specific instrument tailored to assess HRQoL in children with CD [8]. Since then, the CDDUX has been translated, culturally adapted, and validated in several countries, including Spain, Argentina, Brazil, and Morocco, allowing for broader international use and comparison across populations [9, 11-13]. However, research applying the CDDUX in Arab or North African contexts remains scarce. Cultural, dietary, and social differences in these regions may significantly influence how children experience and cope with CD. Therefore, studies based on validated local versions of the CDDUX are essential to better understand these populations' specific challenges and guide targeted interventions.

The objective of this study is to assess the HRQoL of children with CD, using the Moroccan-Arabic version of the CDDUX, developed by Guennouni et al. [13], among patients followed in the pediatric gastroenterology unit of the University Hospital Center (CHU) of Oujda. The study also aims to explore how sociodemographic and clinical factors, including socioeconomic status (SES) and family environment, influence HRQoL in this population, thereby addressing a gap in data specific to the Oriental region of Morocco.

## Materials and methods

Questionnaire

To assess the HRQoL of children and adolescents with CD, we used the CDDUX, a disease-specific questionnaire originally developed and validated in the Netherlands. It comprises 12 items divided into three subdomains: “Diet” (six items), addressing the child's feelings about adhering to a lifelong GFD; “communication” (three items), evaluating how the child feels when discussing or explaining their condition; and “living with CD” (three items), focusing on the child's emotional responses and thoughts related to gluten-containing foods [8].

In addition to the child version (for ages 8-18 years), the CDDUX includes a parallel parent version that assesses the perceived impact of CD on the child's daily life across the same three domains.

Responses are rated on a five-point Likert scale, supported by facial expression illustrations to facilitate comprehension. Higher scores indicate better HRQoL. Raw scores were converted to a 0-100 scale, with the following interpretations: ≤20 (very poor), 21-40 (poor), 41-60 (neutral), 61-80 (good), and >81 (very good) QoL.

We used the Moroccan-Arabic version of the CDDUX, previously validated in a Moroccan pediatric population by Guennouni et al. [13]. Permission to use this version was obtained from the author via email before implementation.

Patients

The Arabic version of the CDDUX questionnaire was administered to all children aged 8 to 18 years who were seen at our center over a 12-month period, as well as to their parents. In total, 49 out of 51 families (96%) agreed to participate in the study.

All patients were followed at the Pediatric Gastroenterology Unit of the University Hospital Center (CHU) of Oujda, Morocco. Diagnoses were confirmed according to the guidelines of the European Society for Paediatric Gastroenterology, Hepatology and Nutrition (ESPGHAN) [14].

The questionnaire also included sociodemographic and economic variables (sex, age, living environment, educational level, marital status, and health insurance coverage), along with medical characteristics (time since diagnosis, recent anti-transglutaminase antibody levels, family history, associated diseases, adherence to the GFD, and its duration).

Statistical analysis

The scores obtained in this study were analyzed using Statistical Package for Social Sciences (SPSS) version 25 (IBM Corp., Armonk, NY). The normality of quantitative data was assessed using the Shapiro-Wilk test, and the homogeneity of variances was evaluated with Levene’s test. Variables that followed a normal distribution were expressed as means and standard deviations (SDs).To assess differences between child and parent scores on the CDDUX, paired samples Student’s t-tests were applied. For comparisons of child-reported CDDUX scores across different sociodemographic, clinical, and socioeconomic categories, independent samples Student’s t-tests were used. A p-value less than 0.05 was considered statistically significant for all analyses. Due to the exploratory nature of the study, no statistical adjustments were made for multiple comparisons. Missing data were minimal and handled by excluding incomplete cases from the relevant analyses.

Ethics and authorization

The study was conducted in accordance with the ethical principles outlined in the Declaration of Helsinki, ensuring patient anonymity and confidentiality. Permission to use the Arabic version of the CDDUX was obtained via email from the corresponding author.

## Results

Study population

Children living in urban areas were the most represented (63%), and the majority were girls (59%). The mean age of the participating children was 12 years and 6 months, with the 12-18 age group being the most represented (59%). The duration of the GFD was categorized into three subgroups: 1-4 years (53%), 5-8 years (25%), and more than 9 years (22%). Among patients with CD, the mean age at diagnosis was 7 years and 6 months (SD ± 4.7). The average parental age was 57 years. Twenty-three percent of the children came from unstable family situations due to divorce or the death of a parent, and 73% had at least one parent with a formal education.

The demographic and clinical characteristics of the 49 participating child-parent pairs are presented in Table 1. At the time of the study, 11 patients had elevated anti-tissue transglutaminase antibody levels, which may indicate poor adherence to the GFD. CD was associated with other chronic conditions in 9 children, with a total of 10 conditions reported, as one child had both hypothyroidism and ichthyosis. These comorbidities included type 1 diabetes, hypothyroidism, asthma, Crohn’s disease, and ichthyosis.

**Table 1:** Socioeconomic, demographic, and clinical characteristics of the study sample (children/parents) (n=49) tTG: Tissue transglutaminase

Characteristic	Category	Number	Percentage
Sex	Male	20	41%
	Female	29	59%
Living area	Urban	30	63%
	Rural	19	37%
Age at interview (years)	Mean = 12.5 years		
Age group	8-11 years	20	40%
	12-18 years	29	59%
Parental marital status	Married	38	77%
	Divorced	7	14%
	One parent deceased	4	9%
Parental education level	Literate	36	73%
	Illiterate	13	27%
Recent tTG antibody test	Positive	11	22%
	Negative	38	78%
Dietary adherence	Poor	2	4%
	Moderate	6	13%
	Good	41	83%
Time since diagnosis	1-4 years	26	53%
	5-8 years	12	25%
	9-12 years	11	22%
Age at diagnosis	Mean = 7.5 years		
Family history of celiac disease	Yes	1	2%
	No	48	98%
Associated conditions	Yes	9	18%
	No	40	82%
Type of associated conditions	Type 1 diabetes	5	10%
	Asthma	1	2%
	Lactose intolerance	1	2%
	Hypothyroidism	1	2%
	Ichthyosis	1	2%
	Crohn’s disease	1	2%

Child-parent concordance

The concordance between the perceptions of children with CD and those of their parents was assessed using scores from the different subscales of the CDDUX (Table 2). Overall, the QoL as perceived by parents was lower than that reported by their children. This difference was statistically significant for the total score, with a mean of 42.46±17.2 in children versus 39.3±14.9 in parents (p=0.01). This suggests that parents generally perceive the impact of CD on daily life as more negative than their children do.

**Table 2:** Assessment of quality of life in 49 child/parent pairs with celiac disease using the Moroccan-Arabic version of the CDDUX CDDUX: Coeliac Disease Dutch Questionnaire

CDDUX	Child score	Standard deviation	Parent score	Standard deviation	t-value	p-value
Having the disease	32.8	19.3	30.1	17.5	t=2.28	0.027
Communication	42.4	16.8	38.2	16	t=2.83	0.007
Gluten-free diet	45	15.5	42.5	14.5	t=1.35	0.184
Total score	42.46	17.2	39.3	14.9	t = 2.68	0.01

The discrepancy was particularly notable in the "communication" subscale, where mean scores were 42.4±16.8 for children and 38.2±16 for parents (p=0.007). This indicates a difference in how the two groups perceive discussions about the disease and the challenges of talking about it with others.

In contrast, for the "GFD" subscale, although parents reported slightly lower scores, the difference was not statistically significant (p=0.184). This may suggest that, with regard to dietary adherence, parents and children share similar perceptions of the associated challenges.

Influence of socioeconomic, demographic, and medical factors

The analysis of socioeconomic, demographic, and medical factors revealed significant variations in QoL among children with CD (Table 3). Age emerged as a key determinant: adolescents (12-18 years) reported significantly higher overall CDDUX scores than younger children (8-11 years), particularly in the subscales having the disease (32.3 vs. 30.5), communication (42.2 vs. 21.1), and GFD (44.3 vs. 20.5), with a significant difference in the total score (p=0.003). No significant sex-related differences were observed (p=0.06).

**Table 3:** Influence of socioeconomic, demographic, and clinical characteristics of study sample (children) on the scores of M-C-DUX (global and subscales) GFD: Gluten-free diet; M-C-DUX: Moroccan version of the Celiac Disease Dutch Questionnaire

Parameter	Category	Having the disease	Communication	GFD	Total	t-value	p-value
Age	8-11 years	30.5	21.1	20.5	24	t=3.13	0.003
	12-18 years	32.3	42.2	44.3	39.6		
Sex	Female	31.3	22.2	37.2	30.2	t=1.91	0.06
	Male	32.4	32.3	38.9	34.5		
Living area	Urban	48.9	41.4	36.3	42.2	t=3.29	0.002
	Rural	20.1	12.7	14.2	15.6		
School attendance	Regular schedule	42.2	41.3	39.7	41	t=1.84	0.07
	Full time	42.3	45.1	38.3	41,9		
Parents' age	>57 years	42.6	44.7	39.5	42.2	t=1.79	0.08
	<57 years	38.6	31.1	41.2	36.9		
Parental status	Deceased/divorced	17.6	22.4	24.1	21.3	t=3.12	0.003
	Stable	39.2	35.8	46.6	40.5		
Socioeconomic level	High	33.8	41.3	49.8	41.6	t=3.85	0.0004
	Low	28.1	24.0	11.3	21.1		
Parental education	Literate	39.6	46.6	59.5	48.5	t=3.47	0.001
	Illiterate	30.4	21.7	28.6	26.9		
Diet duration	<2 years	31.2	40.7	37.9	36.6	t=4.08	0.0001
	>2 years	42.7	51.9	58.4	51		
GFD adherence	Poor	29.9	22.1	19.7	23.9	t=3.26	0.002
	Good	42.2	31.3	42.7	38.7		

The living environment had a marked impact on perceived QoL. Children in rural areas scored significantly lower than those in urban settings, especially in having the disease (20.1 vs. 48.9) and communication (12.7 vs. 41.4), indicating greater challenges in disease management and social integration (p=0.002). Limited access to educational, healthcare, and social resources in rural areas may contribute to these disparities, highlighting the need for targeted interventions in underserved communities.

Family structure also played a critical role. Children from stable families scored significantly higher across all subscales compared to those from disrupted households (e.g., following divorce or bereavement). For instance, in having the disease, scores were 39.2 vs. 17.6, and in GFD, scores were 46.6 vs. 24.1 (p=0.003). Stable environments may provide emotional support and better adherence to dietary management, whereas instability can exacerbate psychological stress and reduce dietary compliance.

SES had a strong influence. Children from higher-income families reported significantly better QoL, particularly in communication (41.3 vs. 24.0) and GFD (49.8 vs. 11.3), with a marked difference in the global score (41.6 vs. 21.1; p=0.0004). Similarly, parental education was associated with better outcomes. Children of educated parents scored significantly higher, especially in communication (46.6 vs. 21.7), GFD (59.5 vs. 28.6), and the total score (48.5 vs. 26.9; p=0.001), suggesting that higher parental education enhances disease understanding and management.

Dietary factors were also significant. Longer adherence to a GFD correlated with better QoL. Children on the diet for more than two years had substantially higher scores in GFD (58.4 vs. 37.9) and communication (51.9 vs. 40.7), reflecting improved adaptation over time (p=0.0001). Additionally, strict adherence to the GFD was linked to better outcomes in all domains, with significantly higher scores in having the disease (42.2 vs. 29.9) and communication (31.3 vs. 22.1; p=0.002).

These findings highlight the profound influence of social, educational, and medical factors on the QoL in children with CD. Sustained and strict dietary adherence, family stability, socioeconomic support, and parental education all contribute to better disease perception and psychosocial well-being.

Variability across sociocultural contexts

**Figure 1:**
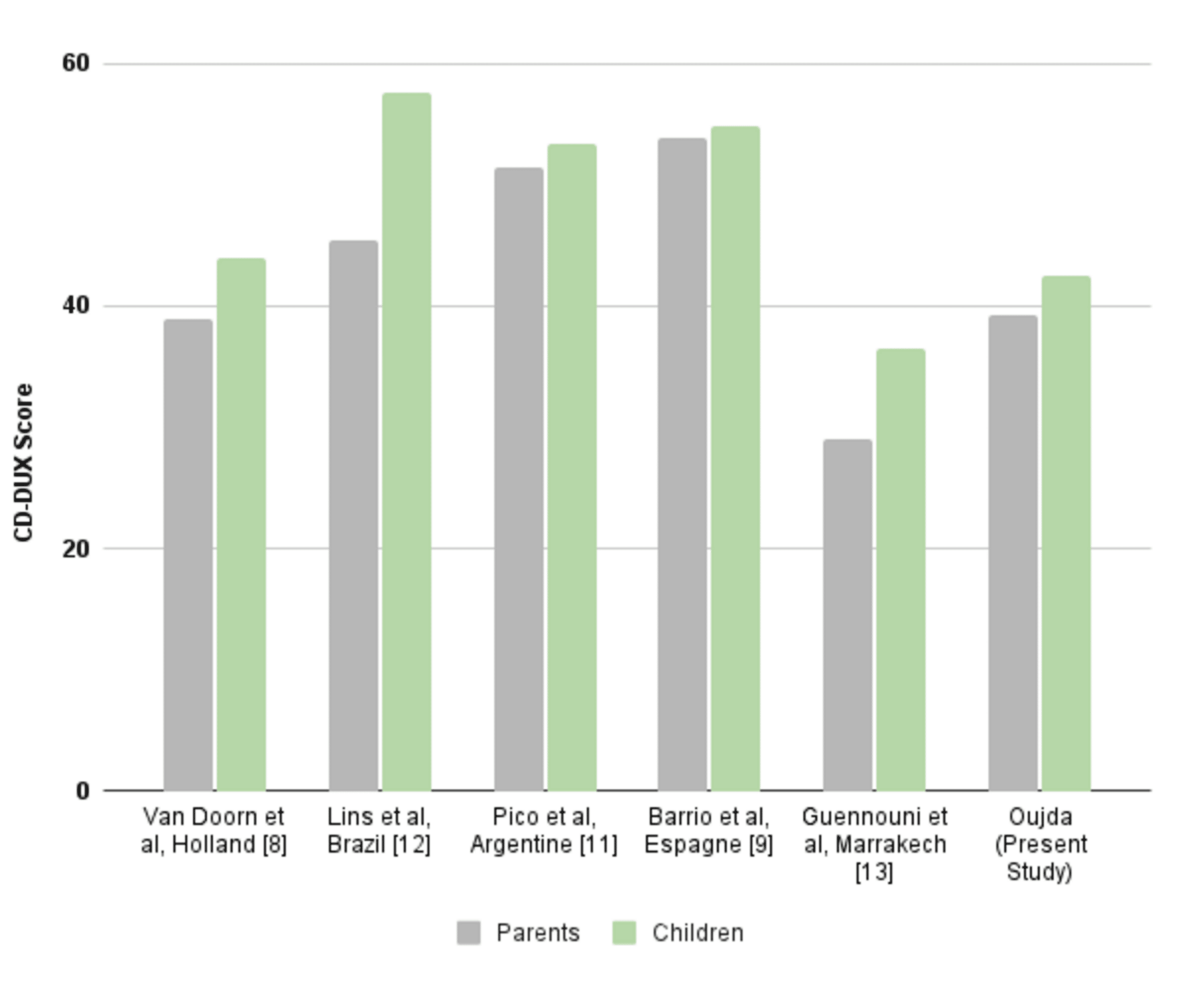
Average CDDUX scores reported by children with CD and their parents in different countries CD: Celiac disease; CDDUX: Coeliac Disease Dutch Questionnaire

A comparative analysis was conducted using CDDUX scores reported in various studies involving children with CD and their parents (Figure 1). These studies, drawn from diverse national populations, provide valuable insights into how cultural, socioeconomic, and geographic contexts may influence perceptions of QoL.

Differences in dietary habits, access to gluten-free products, and social norms can significantly shape the lived experience of CD. This variability highlights the unique challenges families face in different settings and underscores the importance of tailoring management strategies to the specific sociocultural context.

## Discussion

The subjective perception of health status among patients with chronic conditions is increasingly recognized as a critical indicator of overall well-being. Over the past two decades, this dimension of assessment has gained prominence, complementing traditional clinical and biological parameters. Recent research emphasizes the importance of understanding patients’ own perspectives to capture the full impact of chronic diseases not only medically but also in terms of emotional, physical, and social well-being [15].

In the context of CD, evaluating HRQoL has become a key aspect of comprehensive care. It allows clinicians to assess how the disease affects daily life, emotional concerns, comfort, and social interactions, providing a more holistic picture that extends beyond clinical symptoms alone. As such, HRQoL is now regarded as an outcome measure as vital as biological markers [16].

To date, two disease-specific tools have been validated to assess HRQoL in children with CD. The CDDUX questionnaire, originally developed in Dutch and English, has since been adapted into several languages, including Spanish, Portuguese, Farsi, and Argentinian Spanish, to account for cultural variation. CDDUX evaluates physical, emotional, and social aspects of life using child-friendly smiley-based responses, making it simple and effective in pediatric settings. It helps clinicians and researchers better understand the real-life impact of CD and the outcomes of clinical interventions.

The Celiac Disease Pediatric QoL (CDPQoL), validated in the US, offers an alternative specifically designed for American pediatric populations. Both tools provide a comprehensive assessment by integrating the perspectives of both children and their caregivers, highlighting the broader impact of CD on QoL and allowing for tailored therapeutic strategies [8,10].

Individual-level comparisons revealed discrepancies between children’s self-reported QoL and the assessments provided by their parents, echoing findings from previous studies. Parents often tend to rate their children’s QoL more negatively than the children themselves. In other words, they perceive the impact of CD on their child’s well-being as more severe than what the children report when completing the questionnaire independently.

This discrepancy may be attributed to several factors, including differences in symptom perception, emotional awareness, or the ways children experience and cope with their condition, often with greater resilience or without full awareness of the broader implications for their overall well-being. Such differences are not unique to CD; similar patterns have been observed in studies involving other chronic illnesses in pediatric populations. In many cases, parents may underestimate their child’s coping abilities or overinterpret the disease’s negative impact. While such perception differences are expected in younger children, we addressed this by having both children and parents complete the CDDUX. For children around 8 years old, parents helped clarify questions without influencing responses, ensuring better comprehension and more reliable comparisons. It is important to acknowledge limitations in both child and parent reports. Parents may overestimate the disease’s impact due to concern or anxiety, while children might underreport difficulties because of limited emotional awareness or a desire to please. These differences can introduce bias, affecting the accuracy of HRQoL assessments. Future research should incorporate qualitative methods or longitudinal designs to better capture how children’s and families’ perspectives evolve over time. Overall, these findings underscore the need to include children’s own views rather than relying solely on proxy reports when assessing QoL [17,18].

The data also indicate that children who strictly adhere to a GFD report significantly better QoL. These results are consistent with previous findings by Mustalahti, Hallert, and colleagues [15,19]. In this study, adherence was classified based on clinical judgment and patient self-report, with recent anti-transglutaminase antibody levels used to support and corroborate adherence assessment. However, some studies involving long-term treated patients have shown that their subjective well-being does not always reach the levels seen in the general population. Despite ongoing medical management, these patients often report a lower QoL, likely due to persistent symptoms or long-term psychosocial effects [15,20,21].

These observations underscore the critical importance of dietary adherence in managing CD. A strict GFD not only alleviates physical symptoms and prevents long-term complications but also appears to support better psychological adjustment. As a result, children adhering closely to the diet tend to perceive their overall well-being more positively. This finding reinforces the role of dietary compliance as a key factor in improving both the physical and emotional QoL in pediatric CD [22-24].

Several limitations should be considered when interpreting these findings. The relatively small sample size and single-center design may restrict the generalizability of the results. The cross-sectional nature of the study also limits the ability to draw causal inferences. Additionally, reliance on self-reported and proxy assessments may introduce bias, especially given the discrepancies noted between child and parent responses. Future research involving larger, more diverse samples and longitudinal designs is recommended to strengthen and expand these observations.

## Conclusions

Our study supports the systematic assessment of HRQoL in the follow-up care of children with CD, using disease-specific questionnaires. The tendency of parents to rate their children's QoL more negatively than the children themselves raises important concerns regarding the evaluation of chronic illness impact in pediatric populations. These discrepancies may stem from factors such as differing perceptions of symptoms, parental emotional concerns, or the way children experience their condition, often with greater resilience or a different awareness of their limitations.

Our findings highlight the critical importance of incorporating children’s self-reported perspectives to achieve a more accurate and comprehensive understanding of their well-being and disease management. The divergences between parental and child assessments point to the need for a more nuanced and individualized approach to evaluating the QoL in young patients.
